# Comprehensive Evaluation of Multispectral Image Registration Strategies in Heterogenous Agriculture Environment

**DOI:** 10.3390/jimaging10030061

**Published:** 2024-02-29

**Authors:** Shubham Rana, Salvatore Gerbino, Mariano Crimaldi, Valerio Cirillo, Petronia Carillo, Fabrizio Sarghini, Albino Maggio

**Affiliations:** 1 Department of Engineering, University of Campania “L. Vanvitelli”, Via Roma 29, 81031 Aversa, Italy; shubham.rana@unicampania.it; 2 Department of Agricultural Sciences, University of Naples “Federico II”, Via Università 100, 80055 Naples, Italy; mariano.crimaldi@unina.it (M.C.); valerio.cirillo@unina.it (V.C.); fabrizio.sarghini@unina.it (F.S.); albino.maggio@unina.it (A.M.); 3 Department of Biological and Pharmaceutical Environmental Sciences and Technologies, University of Campania “L. Vanvitelli”, Via Antonio Vivaldi, 43, 81100 Caserta, Italy; petronia.carillo@unicampania.it

**Keywords:** binary mask, homography matrix, masked pixels, MS (multispectral), SIFT (scale-invariant feature transform)

## Abstract

This article is focused on the comprehensive evaluation of alleyways to scale-invariant feature transform (SIFT) and random sample consensus (RANSAC) based multispectral (MS) image registration. In this paper, the idea is to extensively evaluate three such SIFT- and RANSAC-based registration approaches over a heterogenous mix containing *Triticum aestivum* crop and *Raphanus raphanistrum* weed. The first method is based on the application of a homography matrix, derived during the registration of MS images on spatial coordinates of individual annotations to achieve spatial realignment. The second method is based on the registration of binary masks derived from the ground truth of individual spectral channels. The third method is based on the registration of only the masked pixels of interest across the respective spectral channels. It was found that the MS image registration technique based on the registration of binary masks derived from the manually segmented images exhibited the highest accuracy, followed by the technique involving registration of masked pixels, and lastly, registration based on the spatial realignment of annotations. Among automatically segmented images, the technique based on the registration of automatically predicted mask instances exhibited higher accuracy than the technique based on the registration of masked pixels. In the ground truth images, the annotations performed through the near-infrared channel were found to have a higher accuracy, followed by green, blue, and red spectral channels. Among the automatically segmented images, the accuracy of the blue channel was observed to exhibit a higher accuracy, followed by the green, near-infrared, and red channels. At the individual instance level, the registration based on binary masks depicted the highest accuracy in the green channel, followed by the method based on the registration of masked pixels in the red channel, and lastly, the method based on the spatial realignment of annotations in the green channel. The instance detection of wild radish with YOLOv8l-seg was observed at a mAP@0.5 of 92.11% and a segmentation accuracy of 98% towards segmenting its binary mask instances.

## 1. Introduction

The primary challenge faced in the registration of images with distinct spectra during close-range imaging using multispectral (MS) cameras is misalignment among different spectral channels [1]. Subsequent tasks such as information identification, extraction, and feature detection become more challenging because of these distortions [2,3,4,5]. An ideal solution to this issue would be a reliable, accurate, and simple-to-use registration approach that accounts for the disparity in perspective of individual sensors. A much-needed addendum for improving such misalignment is intra-subject registration, which involves fine-tuning annotation coordinates to overlap with each other, thereby achieving the registration of the pixels of interest.

In contrast to a single-lens imaging system, multi-lens imaging systems may comprise one or more cameras that utilize multiple image sensors and lenses to capture individual spectral ranges. Nevertheless, the original MS images from a multi-lens imaging system present significant band misregistration errors such as lens distortion, varied positions, and viewing angles of every lens [6]. These distortions and ghosting effects in the original MS images lead to geometrical distortions, which must be rectified through band co-registration for accurate spectral analysis in remote sensing [7]. In our experiment, MicaSense RedEdge-M was used to acquire close-range agricultural images. The MicaSense RedEdge-M features a multi-lens design that allows users to capture specific narrowband spectral data using individual lenses with band-pass filters [8]. However, registering these images can be challenging due to differences in scale, orientation, and perspective.

Registering MS images is useful for change detection, where changes in shape, size, and area can be analyzed from multiple spectral channels collected over a period. By incorporating additional spectral information, it is possible to enhance the discrimination of different objects and features within images [9,10,11]. This makes it possible to identify and classify crops more accurately, which improves the process of image segmentation [12]. Close-range MS imaging involves capturing images of the same field within sub-meter distances on crops using different spectral bands. A single-lens imaging system employs one image sensor which can obtain multiple spectral bands by either altering the sensor filter [13] or employing the Fabry–Perot interferometer (FPI) technique [14].

David Lowe devised the scale-invariant feature transform (SIFT) algorithm in 1999, which was employed in computer vision to detect, describe, and match features in images [15,16]. One of its applications includes aligning two or more images through the identification and matching of corresponding features or keypoints in the images [17,18]. The accuracy of SIFT has been established to be one of the highest among feature detector descriptor algorithms for scale and rotation variations. It also has greater accuracy for image rotation than other algorithms and has been determined to be the most precise algorithm across all geometric transformations [19,20,21,22]. This technique can be particularly useful for improving MS image registration and alignment while utilizing binary masks, which can be used to segment and extract specific regions of interest from MS images.

This paper provides a certain reference to other scholars to advance their research on weed detection algorithms based on computer vision and achieve intelligent weed control and related areas of research and application [23]. There has been a similar work based on proximal sensing and feature-based MS image registration following different geometric transformations in plants imaged in a greenhouse environment using the same sensor [24]. Another experiment based on Parrot Sequoia MS images performed co-registration without assumptions based on scene structure and just required dense matching between two spectrally similar channels [7]. The weed *Raphanus Raphanistrum*, also known as wild radish, belongs to the family of *Brassicaceae*, whose infestation in the cropping of wheat is a subject of research interest [25,26]. Wild radish poses a significant threat to winter crops, including wheat, as it is one of the most aggressive and competitive broad-leaved weeds. Despite the availability of various chemical and non-chemical control methods, the prevalence and spread of wild radish seem to be on the rise [27]. It is a highly troublesome, aggressively invasive, and enduring weed. Its abundant seed generation, harmful effects on other crops, herbicidal resistance, seed inactivity, and variability in appearance and genetics make it challenging to control [28,29,30]. The excessive dependence on herbicides for weed control has led to its resistance to these chemicals. Therefore, incorporating computer vision strategies based on detection and segmentation is crucial for improving its management [23].

We are specifically interested in the evaluation of three different registration strategies based on SIFT and RANSAC over close-ranged sub-meter MS images to evaluate intra-subject registration. This paper compares these methods through a qualitative and quantitative perspective at a spectral level. The datasets of weed identification and detection and leaf classification are summarized, and the problems faced in field weed detection under different conditions are analyzed.

## 2. Materials and Methods

Our work focuses on three different approaches to the evaluation of MS image registration. The first method is based on the registration of individual spectral channels to a reference spectral channel using SIFT and random sample consensus (RANSAC) [31], at a time, and utilizes the homography matrix, derived during registration for the realignment of all the annotations performed across respective spectral channels. This means that spatial readjustment is performed on the coordinates of the annotations across the four spectral channels using the homography matrices obtained during the registration of every spectral band. The second method is based on segmentation-based registration [32] of binary mask images obtained from the annotated ground truth, which are registered using SIFT and RANSAC. In this method, every binary mask image corresponding to a spectral channel was registered to the binary mask image of the reference spectral channel. The third method is based on the mask-based registration of the pixels of interest derived from the annotated ground truth. In this method, only the masked pixels are registered using SIFT and RANSAC.

Interestingly, there has been a limitation of studies in the evaluation of the impact of segmentation over subsequent registration [32]. In the context of realignment of annotations, registration of binary mask images, as well as registration of pixels of interest derived from the annotated ground truth to overall achieve the registration of MS images, SIFT was used to detect and extract feature points, and then RANSAC was used to estimate the transformation between the two sets of feature points.

A state-of-the-art instance segmentation model, YOLACT (You Only Look at Coefficients) [33], was chosen to train the annotated MS images for evaluation of the predicted masks. YOLACT can quickly and accurately segment instances in images by dividing the task into two parallel subtasks: generating prototype masks and predicting mask coefficients. YOLACT utilizes these coefficients to linearly weight the prototype masks, resulting in high-quality instance masks.

Registration of the MS images based on pixels of interest masked through predicted binary mask images post-trained with YOLOv8 provides us room for comparative evaluation with manually segmented images and registered pixels of interest using the three methods described above. In our experiments, the overall emphasis is laid on exploiting these multiple ways of applying SIFT and RANSAC towards the registration of MS images based on annotated pixels of interest masked through binary masks across different spectral channels. The goal is to comprehensively evaluate the registration of annotated pixels of interest across manually and automatically segmented MS images.

The perspective homographic transformation was used in our experiment as images containing objects of interest appear misaligned across different spectral channels due to foreshortening and inter-sensor separation [34,35]. An affine transformation usually has six degrees of freedom and can perform translation, rotation, scaling, and skewing operations. It preserves parallel lines, ratios of distances along parallel lines, and angles [36,37]. A perspective transformation has eight degrees of freedom and can model projective distortions [38,39].It can perform all the operations of an affine transformation plus account for distortion caused by changes in viewpoint or camera position. If the images have significant differences in perspective or viewpoint, or if the task requires accounting for projective distortions, a perspective realignment is likely to be a better choice [40,41,42]. If the images only need to be translated, rotated, or scaled, an affine transformation may be sufficient.

**Figure 1:**
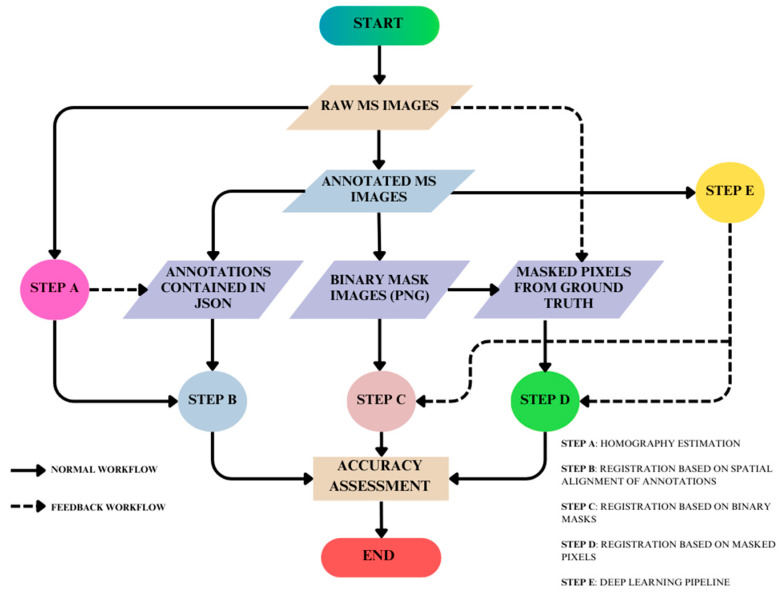
Overall methodology comprising of individual sub-techniques.

The overall methodology (Figure 1) is a holistic picture of the three sub-methodologies adopted in this research. It begins with MS image acquisition over which mask annotations are performed using the VGG (Visual Geometry Group) VIA (VGG Image Annotator). This dataset is further classified into three categories. The first cluster consists of the annotated MS images with the spatial coordinates of wild radish annotations contained in JSON (JavaScript Object Notation). The second cluster consists of the binary mask images. The third cluster consists of the pixels of interest masked using the raw MS images and the binary mask images. In Step A, the homography matrix is calculated using raw MS images, which is then used to align the spatial coordinates of the annotations contained in the JSON (JavaScript Object Notation) in Step B. Therefore, Step B is the registration of MS images based on the spatial realignment of annotations. Step C is the registration of MS images based on the registration of binary mask images (.PNG format), wherein the input comes from the second cluster of the dataset. Step D is the registration of MS images based on the registration of masked pixels, wherein the input comes from the third cluster of datasets. Step E is a deep learning framework in which the annotated MS images are trained over the YOLOv8l-seg model to automatically predict wild radish masks. These automatic predictions are then subjected to morphological erosion before being provided as input to Step C and Step D. These images are further masked before being fed back to Step D. Finally, an accuracy assessment is performed over the binary mask outputs coming from Steps B, C, and D to evaluate the quality of registration achieved with these methods, and overall, compare manual and automatic segmentation.

### 2.1. Dataset

#### 2.1.1. Image Acquisition

**Figure 2:**
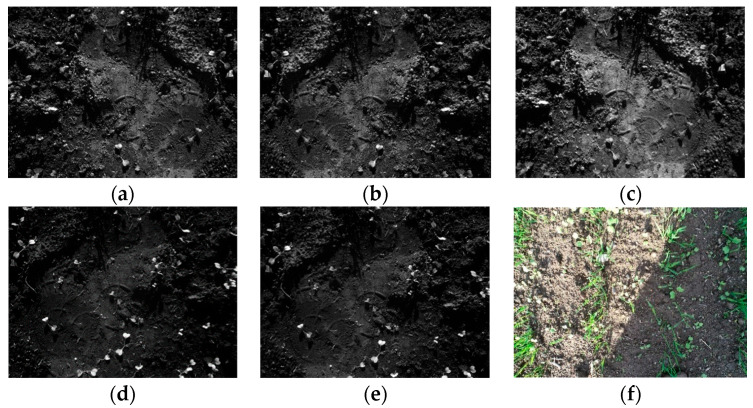
(**a**–**e**) Blue, green, red, near-infrared, and RedEdge instances of *R. raphanistrum* weed; (**f**) RGB instance of heterogenous mix of *Triticum aestivum* crop and *R. raphanistrum* weed.

**Figure 3:**
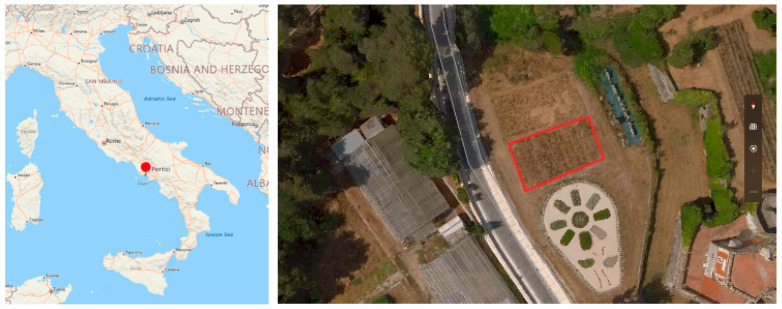
Locational information of the bread wheat farm in Department of Agronomy, University of Napoli Federico II (study area marked in red polygon). Source: Imagery @2020 Airbus, Maxar Technologies, Google Earth.

The dataset (Figure 2a–e) consists of 80 raw and 80 labeled MS images containing a mix of bread wheat and wild radish. Manual acquisition of MS images was carried out over an experimental farm situated in the Department of Agricultural Sciences, University of Napoli Federico II, Portici, Italy (Figure 3: appx. lat.: 40°48′52.1139″ N, long.: 14°20′48.4242″ E; elevation 80.422 m above the sea level). The acquisition was performed on 18 January 2022 using a MicaSense RedEdge-M camera (Figure 3). The sensor was kept at 1 m using a gimble for the acquisition of images. For each scene, the camera saves five files, one per spectral channel, resulting in five files per scene. So, there are 16 image scenes captured over five spectral channels, thereby totaling up to 80 images. The resolution of the images is 1280 × 960 pixels with a radiometric resolution of 8 bits. The war and annotated dataset can be accessed at [43]. The sensor specifications are illustrated in Table 1.

#### 2.1.2. Annotation of MS Images

Pixel annotations were performed over the raw MS images using VIA (VGG Image Annotator, https://gitlab.com/vgg/via, accessed on 29 January 2024), which is open-source labeling software that was developed at the Visual Geometry Group (VGG), University of Oxford [44]. This software is designed to operate independently, without necessitating any form of installation or configuration on a personal computer. The annotations were subsequently exported in the form of JavaScript Object Notation (JSON).

#### 2.1.3. Software

The codes to perform SIFT- and RANSAC-based image registration, extraction of homography matrices, registering images based on spatial realignment of annotations, registering binary masks to achieve image registration, registering pixels of interest to achieve image registration, extraction of individual and semantic segmentation masks based on annotations, object detection and segmentation based on training YOLOv8l-seg network, prediction of mask instances using trained weights, and morphological dilation of segmentation masks were scripted in Python v3.11.

### 2.2. Homography Matrix Estimation: Step A

#### 2.2.1. Keypoint Detection and Feature Extraction

**Figure 4:**
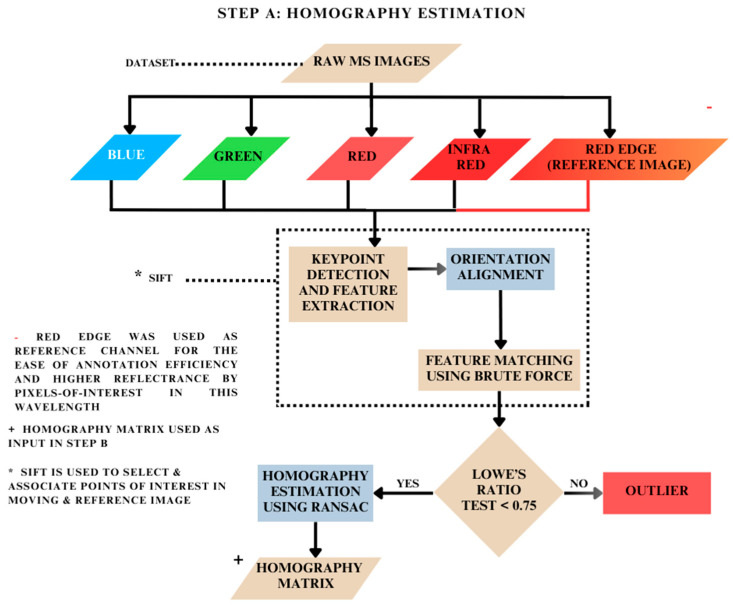
Methodology for homography estimation.

When estimating the homography matrix using RANSAC and the direct linear transform (DLT), the first step is to detect keypoints and extract features from the images [39,45,46]. This is carried out using the SIFT algorithm, which finds keypoints in an image by looking for locations that are invariant to changes in scale and orientation (Figure 4). These locations are identified by finding extrema in the difference-of-Gaussian function applied to the image at multiple scales. Once the keypoints are detected, their orientation is assigned based on the dominant gradient direction in the local neighborhood of the keypoints. This ensures that the keypoint descriptor is invariant to rotation [16]. Once the keypoints are detected, the next step is to match features from these keypoints.

#### 2.2.2. Feature Matching

After the features were extracted from two images, the next step was to match the features between them. This was achieved by comparing the descriptors of keypoints in the moving image with those in the reference image (Figure 4). Brute-force matcher exhaustively compares every feature descriptor in one image with every feature descriptor in the other image [47,48]. Once the keypoints and their features were matched, a set of corresponding point pairs was obtained. These point pairs were further evaluated for filtering and subsequently used to estimate the homography matrix between the two images.

#### 2.2.3. Ratio Test and Filtering

To remove the incorrect matches, Lowe’s ratio test was used. This test involves finding the two best-matching descriptors in the second image for each descriptor in the first image based on the smallest distance [49]. The ratio of the distances between the best and second-best matches was found to be good below a certain threshold of 0.75. Beyond this threshold, the matches were filtered out as they were found to be ambiguous. Applying this test, we were left with a set of potential correspondences between the two images (Figure 4). However, theoretically, these correspondences may still contain outliers or mismatches [16].

#### 2.2.4. Homography Estimation

RANSAC is an algorithm used to estimate the homography matrix that maps the coordinates of keypoints in one image to their corresponding coordinates in another image. The aim of RANSAC is to identify a set of matches that are most likely to be correct and use them to estimate the homography matrix. It works together with the direct linear transform to calculate the homography matrix by eliminating incorrect point correspondences that could lead to an inaccurate outcome [50]. This is necessary to overcome incorrect matches due to noise or other factors. RANSAC works by iteratively selecting a random subset of matches, estimating a tentative homography matrix based on these matches, and then testing the remaining matches against this matrix to identify inliers [31]. In this way, a tentative matrix with the most inliers is chosen as the final homography matrix. In our experiment, we typically set the error threshold to 0.75 when calculating the distance between matched keypoints, as matches that are farther apart were found to be outliers (Figure 4). Additionally, we set the number of iterations to be proportional to the number of matches, as more matches require more iterations to find the best set of inliers. Once the final homography matrix was estimated, it was used to warp the moving image so that it aligned with the reference image.

### 2.3. Spatial Realignment of Pixel Annotations: Step B

**Figure 5:**
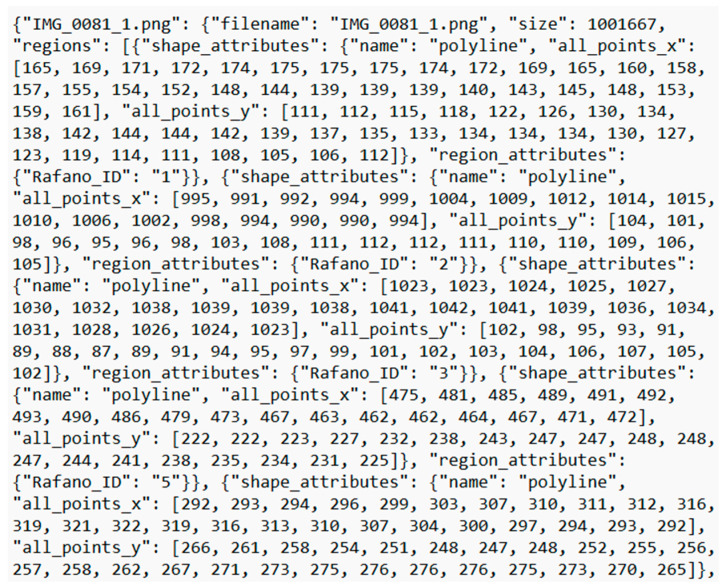
Structure of JSON file.

**Figure 6:**
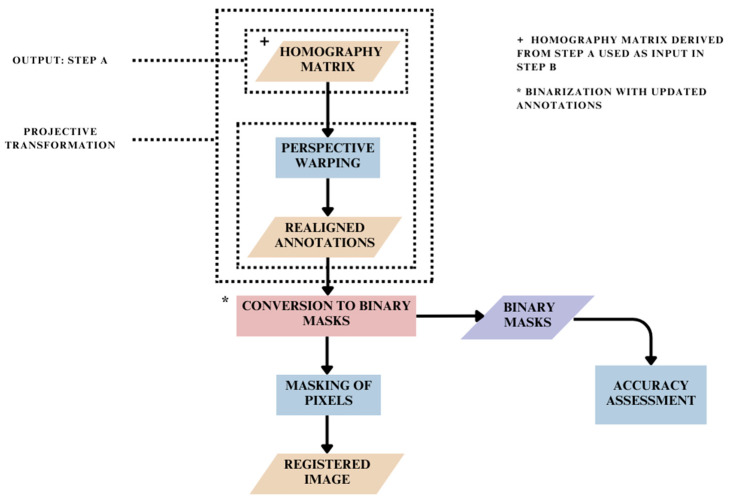
Methodology for MS image registration based on spatial realignment of pixel annotations.

After calculation of the homography matrices (Figure 4) for each spectral channel in relation to its RedEdge counterpart, these matrices were used to realign the spatial coordinates of each annotation across all four spectral channels. In this manner, the spatial attributes of the annotations contained in the ‘all_points_x’ and ‘all_points_y’ fields are updated in the JSON file (Figure 5). Subsequently, a new set of binary masks was obtained using the spatially updated annotations and an updated JSON file. In this way, registration of MS images based on re-referencing of updated annotation coordinates was achieved (Figure 6).

### 2.4. Registration Based on Binary Mask Images: Step C

**Figure 7:**
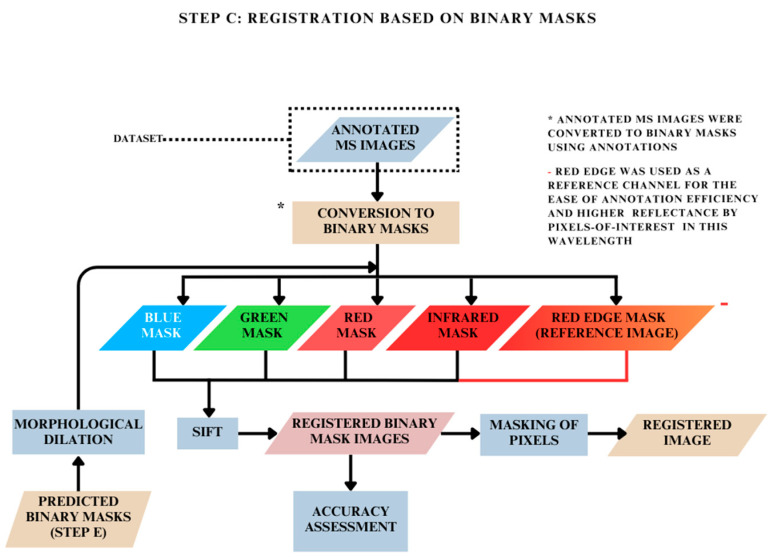
Methodology for MS image registration based on registration of binary masks.

In this method, firstly, the binary masks were obtained using annotated MS images. Subsequently, the features were detected and matched between the binary masks of each spectral channel and its corresponding RedEdge conjugates across all image scenes using SIFT and RANSAC (Figure 4). In this step, the keypoint estimation and feature detection were carried out for the binary mask images across all four spectral channels. The correspondences found here were then used to estimate the transformation required to align the masks, thereby registering the binary mask images (Figure 7).

### 2.5. Registration Based on Masked Pixels: Step D

**Figure 8:**
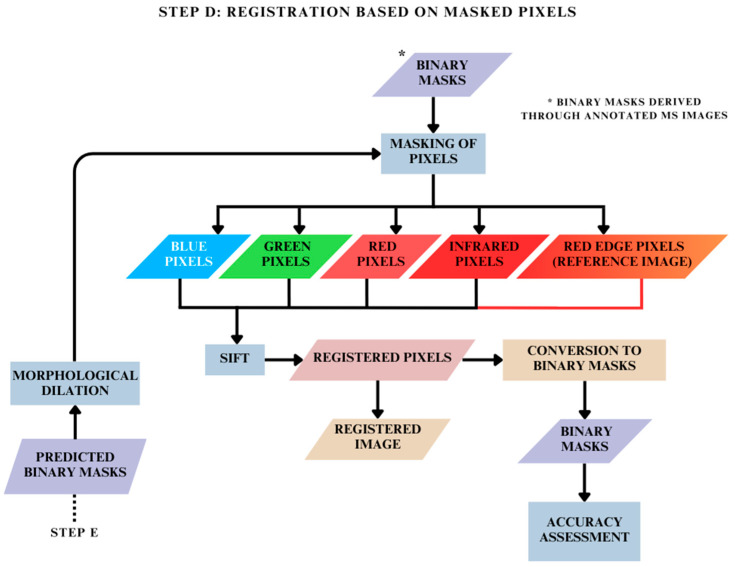
Methodology for MS image registration based on registration of masked pixels.

The pixels of interest were masked using the binary masks obtained after conversion in Step C focused on image registration based on binary masks and raw MS images of the dataset (Figure 7). Subsequently, only these non-zero pixels of interest were registered using SIFT and RANSAC methods as described in Figure 4. The implementation is described in Figure 8.

### 2.6. Deep Learning Pipeline for Training Annotated MS Images: Step E

A group of deep learning models created for object recognition is represented by the YOLO (You Only Look Once) series. The most recent version, YOLOv8, was designed by the same team as YOLOv5, and it keeps the same architectural design. The YOLOv8 network performs additional tasks apart from object recognition and tracking, particularly instance segmentation, image categorization, and keypoint detection [51]. YOLOv8 supports five distinct model sizes (n, s, m, l, and x), each of which increases in depth and width from left to right. YOLOv8l-seg was chosen for the reason of optimum compatibility with our available computational resources. The YOLOv8l-seg network’s design is influenced by the YOLACT network’s principles [33], allowing it to segment objects in real time while maintaining a high segment mean average precision. In this study, the training and prediction of wild radish instances were carried out using the YOLOv8l-seg model.

**Figure 9:**
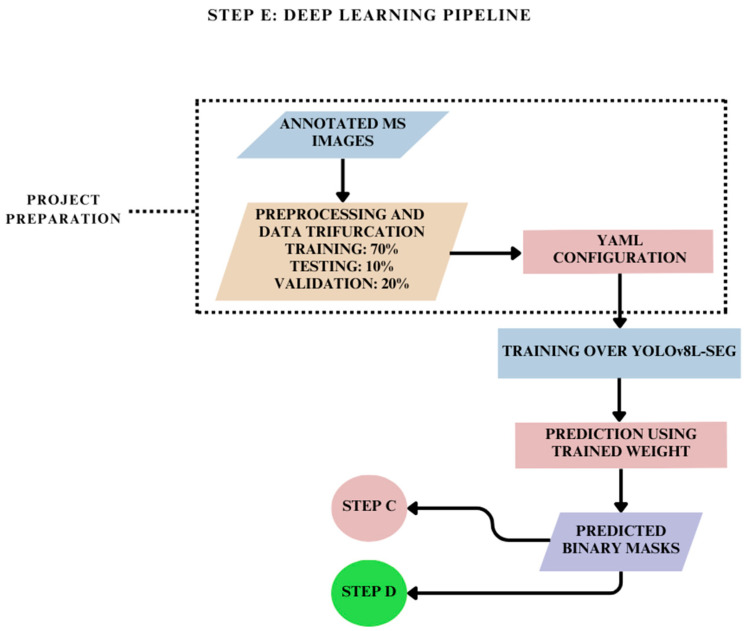
Methodology for training annotated MS images towards instance segmentation.

The dataset was prepared to allot 60 images for training, 8 for testing and 12 for validation. Subsequently, a YAML configuration file was created to associate the relationship between training data, annotations, and weights. A configuration of 200 epochs was chosen for training. The best training weight obtained after training was used to predict the mask instances, which were subsequently fed to Steps C and D (Figure 9) of the overall methodology.

### 2.7. Morphological Dilation of Predicted Masks

The predicted instance masks were now subjected to morphological dilation, which is a technique that enlarges bright regions and shrinks dark regions. Since dilation enhances the size and shape of objects in a binary image. The degree and direction of this enhancement depend on the size and shape of the structuring element. It was used to increase the radius of segmentation [52] so that the pixels of interest are adequately masked for registration purposes.

### 2.8. Registration of Predicted Masks

The predicted and dilated instance masks obtained in Section 2.10 across the four spectral channels from the testing and validation datasets are now registered to their respective binary RedEdge conjugate across all image scenes using SIFT and RANSAC. In this step, the keypoint estimation and feature detection were carried out among the predicted and trained datasets. The correspondences found here were then used to estimate the transformation required to align the predicted masks, thereby registering them.

### 2.9. Registration of Masked Pixels from Predicted Masks

The predicted and dilated instance masks obtained in Section 2.10 across the four spectral channels from the testing and validation datasets are now used for masking the pixels of interest over the raw MS images (Step D). Subsequently, these non-zero pixels are registered using the SIFT and RANSAC to alter the spatial coordinates of the pixels throughout the four spectral channels with respect to the RedEdge channel.

### 2.10. Accuracy Assessment

#### 2.10.1. Intersection over Union (IoU)

Intersection over union is used for assessing the accuracy of the image registration process, which involves the alignment of two images or more with one another. It is based on the measure of similarity in which the intersections of the two sets of pixels are divided by the union of the same two sets of pixels [53]. In the context of the registration of images, the *IoU* (Equation (1)) can be used for measuring the overlaps between the images registered. When two images have been registered, they should ideally have a large overlap, i.e., the same structure or feature should be found in both. The *IoU* provides measurable measures of the overlap and may be used as a measure of the quality of registrations [54]. Specifically, it can be used to evaluate the accuracy of the registration by comparing the registered image to a ground truth or reference image. The *IoU* between the registered image and the ground truth image can be computed, and a high *IoU* value indicates that the registration is accurate. However, *IoU* is often preferred because it provides a more intuitive measure of overlap between the registered images. Mathematically, it is calculated as follows [55]:

(1) $$IoU=\frac{\left|A\;\cap \;B\right|}{\left|A\;\cup \;B\right|}=\frac{TP}{TP+FP+FN}$$

where *A* and *B* are binary image masks, *TP* indicates true positive, *FN* indicates false negative, and *FP* signifies false positive.

#### 2.10.2. Normalized Coefficient of Correlation (NCC)

The normalized coefficient of correlation is based on the measure of the similarity of two images being recorded at the same time. It is based on the statistical measure of the linear relation between the pixel intensity values in the image [56]. The normalized coefficient of correlation, also referred to as a normalized correlation coefficient, measures the relative similarity of two images, by calculating the correlation of the intensity values of the two images. However, it is not considered by the difference between the mean values and the standard deviations of intensity values [57,58].

The normalized correlation coefficient takes these differences into account by normalizing the coefficient of cross-correlation between the two image images and the product corresponding to the average deviation of the values of intensity (Equation (2)). This normalization allows the coefficients of correlation to range from −1 to 1, where closer to 1 indicates a greater degree of similarities between two images. Mathematically, it is calculated as follows:

(2) $$\delta_{c}\left(i_{1},i_{2}\right)=\frac{i_{1}^{T}i_{2}}{∥i_{1}∥\;∥i_{2}∥}$$

where $\delta_{c}$ represents cosine similarity measure based matching score of vectors i_1_ and i_2_, and ||. || represents the norm operator.

#### 2.10.3. YOLOv8l-Seg Evaluation Metrics

The YOLOv8l-seg’s ability to accurately identify instances within an image was assessed using the average precision (*AP*) metric, as outlined in the MS COCO challenges [59]. *AP* is determined by the area under the precision (*P*) and recall (*R*) curves (Equations (3) and (4), respectively), and it is typically computed for each class within an image and then averaged to yield the mean average precision (*mAP*). In this study, as the wild radish class is the sole instance of interest, the *AP* can be considered equivalent to the *mAP* (Equation (5)). The *F*1 score is computed as the harmonic mean of precision and recall, and it demonstrates how the model achieves a balance between accurate detection and thorough coverage. When evaluating segmentation tasks, precision (*P*), recall (*R*), *AP*, and *F*1 *score* are calculated as follows [60,61]:

(3) $$P=\frac{TP}{TP+FP}$$

(4) $$R=\frac{TP}{TP+FN}$$

(5) $$mAP=\int_{0}^{1}P\left(R\right)dR$$

(6) $$F1Score=2\times \frac{P\times R}{P+R}$$

where *TP* (true positive) represents the number of accurate predictions in all pixels, *FP* (false positive) signifies the number of incorrect predictions in all pixels, and *FN* (false negative) indicates the number of pixels that were not identified as part of the corresponding instance. The evaluation metrics, mean average precision (*mAP*), particularly *mAP*_50_ and *mAP*_95_ are distinguished by the intersection over union (IoU) between predicted masks and ground-truth.

Another metric is a confusion matrix, which is a square matrix containing information about the actual and predicted classifications carried out using a classification model. It is a tool used in the field of machine learning to evaluate the performance of a classification algorithm. It provides a summary of the predictions made by a model on a set of test data for which the true values are known [62].

## 3. Results

### 3.1. Evaluation of MS Image Registration Quality

**Figure 10:**
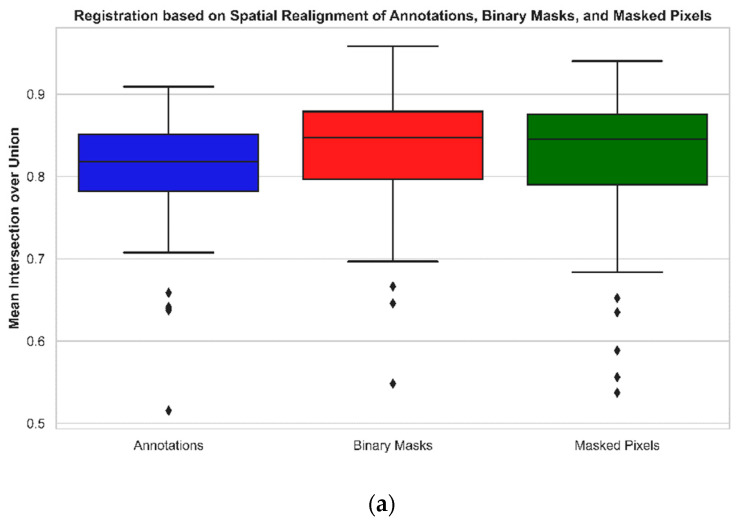

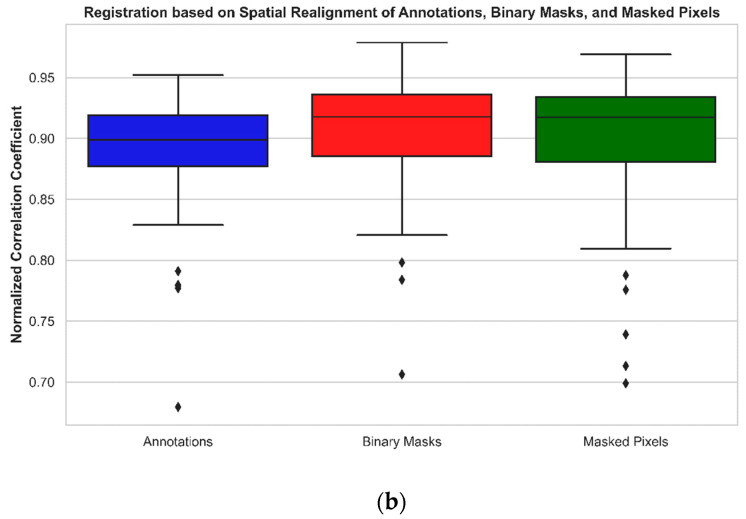
Metrics represented through 2D boxplots towards comparison of registration based on spatial realignment of annotations, binary masks, and masked pixels. (**a**) Mean Intersection over union for annotations, binary masks, and masked pixels; (**b**) normalized correlation coefficient for annotations, binary masks, and masked pixels.

The binary masks obtained in Section 2.3, Section 2.4 and Section 2.5 were tested over metrics such as IoU and NCC to evaluate the MS image registration quality achieved through three techniques: spatial realignment of annotations, registration based on binary masks, and registration based on masked pixels (Table 2). Figure 10 represents two-dimensional boxplots of the three techniques containing samples from 80 annotated images across blue, green, red, near-infrared, and RedEdge spectral channels. The boxplot in Figure 10a shows that the mean IoU for the registration based on binary masks is the highest, recording a mean value of 0.8314 in comparison to the mean IoU observed for the registration based on masked pixels and spatial realignment of annotations, which are 0.8183 and 0.8055, respectively. There are three outliers observed in the case of registration based on binary masks with two outliers occurring in the range of 0.6–0.7 and only one outlier occurring in the range of 0.5–0.6. In the case of registration based on masked pixels, a total of five outliers are observed, with two outliers occurring in the range of 0.6–0.7 and the remaining three outliers occurring in the range of 0.5–0.6. However, in the case of registration based on the spatial realignment of annotations, five outliers are observed, with four outliers occurring in the range of 0.6–0.7 and one outlier occurring in the range of 0.5–0.6. This demonstrates that the registration method based on binary masks performed the best, followed by the registration method based on masked pixels, and lastly, the registration method based on spatial realignment of annotations. A tabular representation (Table 2) of the mean IoU across the above-mentioned methods is presented below.

The boxplot in Figure 10b shows that the mean NCC for the registration based on binary masks is the highest, recording a mean value of 0.9059 in comparison to the mean NCC observed for the registration applied on masked pixels and spatial realignment of annotations, which are 0.8971 and 0.8900, respectively. There are three outliers observed in the case of registration based on binary masks with two outliers occurring in the range of 0.75–0.8 and only one outlier occurring in the range of 0.7–0.75. In the case of registration based on masked pixels, a total of five outliers are observed, with two outliers occurring in the range of 0.75–0.8 and the remaining three outliers occurring in the range of 0.7–0.75. However, in the case of registration based on spatial realignment of annotations, five outliers are observed, with four outliers occurring in the range of 0.75–0.8 and the last outlier observed below 0.7. This, again, demonstrates that the registration method based on binary masks performed the best, followed by the registration method based on masked pixels, and lastly, the registration method based on spatial realignment of annotations. Table 2 presents the mean NCC across the above-mentioned methods.

### 3.2. Evaluation of Registration Quality at Spectral Level

The datasets obtained in Section 2.3, Section 2.4 and Section 2.5 were tested to evaluate registration quality at the spectral level. Table 3 is a behavioral exhibition of annotation quality observed across different wavelengths, i.e., blue, green, red, and near-infrared before registration was performed with respect to their conjugate RedEdge pairs, the reference. It was observed that the near-infrared channel exhibited the best quality of registration in comparison to other spectra over the metrics IoU and NCC. The registration method based on binary masks fared the best in comparison to the ones based on masked pixels and spatial realignment of annotations, thereby recording a mean IoU of 0.8726 and a mean NCC of 0.9170.

### 3.3. Evaluation of Registration Quality at Instance Level

The annotations obtained in Section 2.1.2 were utilized to generate individual wild radish instance masks. Subsequently, these masks across all wavelengths were cloned for evaluation using the three registration methods described earlier. The quantitative behavior (Table 4) of these instance masks across different spectral channels, i.e., blue, green, red, and near-infrared while being registered with their conjugate RedEdge instance pairs, the reference. It was observed that the green channel exhibited the best quality of registration for individual instances as far as registration was performed using binary masks where a mean IoU of 0.9339 and a mean NCC of 0.9668 were recorded. The green channel also exhibited the best performance in the category of registration based on spatial realignment of instance annotations, recording a mean IoU of 0.8969 and a mean NCC of 0.9457 as compared to other spectral channels. However, the red channel recorded the best intra-category performance with a mean IoU of 0.9159 and a mean NCC of 0.9573 in the category of registration based on masked instance pixels. Spectrally, the red channel recorded the least standard deviation of 0.013 for mean IoU, and 0.0118 for mean NCC. Overall, the best registration method for individual instances was observed to be the registration based on binary masks.

### 3.4. Evaluation of Overall Registration Quality for Predicted Instances

The predicted mask instances, obtained from the testing and validation datasets after training the YOLOv8l-seg model on annotated MS images (as described in Section 2.6), were subjected to morphological dilation (Section 2.7) before being registered using the methods described in Section 2.4 and Section 2.5. Table 5 is a holistic representation of registration performances based on predicted binary masks and predicted-and-masked pixels. It was observed that the registration based on predicted binary masks performed marginally better, thereby recording a mean IoU of 0.7188 and a mean NCC of 0.8099. However, the mean IoU for registration based on predicted-and-masked pixels was recorded to be 0.7185 with a mean NCC of 0.8016.

### 3.5. Evaluation of Registration Quality for Predicted Instances across Individual Spectral Channels

The testing and validation datasets containing predicted mask instances (Section 2.6) were then evaluated at the spectral level. Table 6 represents the performance of previously described registration methods applied on predicted binary masks and predicted-and-masked pixels. It was observed that the registration based on binary masks performed the best in the blue channel, recording a mean IoU of 0.7481 and a mean NCC of 0.8525. However, the mean IoU and mean NCC observed with registration based on predicted-and-masked pixels were recorded to be 0.7378 and 0.8462, respectively, being the highest for the green channel. Overall, the registration based on predicted binary masks performed better than the registration based on predicted-and-masked pixels, thereby recording a cumulative mean IoU of 0.7195 and mean NCC of 0.8336.

### 3.6. Error Map

**Figure 11:**
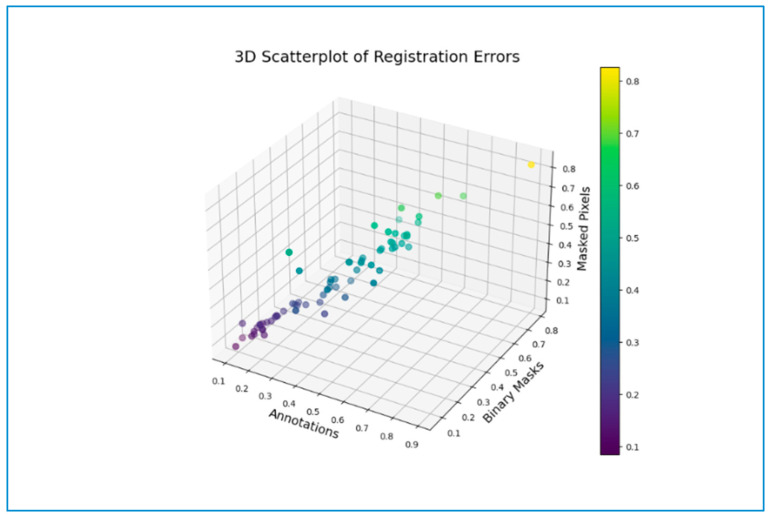
3D scatterplot for comparison of registration errors observed across registration methods based on spatial realignment of annotations, binary masks, and masked pixels.

Figure 11 represents a 3-dimensional scatter plot depicting the error percentage of non-overlapping pixels across the three registration approaches under study. The lowest bias was observed in the registration applied on binary masks with a recorded mean value of 0.3292. However, the outliers in the case of registration applied to masked pixels and annotations were observed to be 0.3526 and 0.3616, respectively. Therefore, the registration technique based on spatial realignment of annotations fared as the least accurate as compared to the other two. In the registration based on automatically segmented binary mask instances, the mean error of non-overlapping annotations after registration was observed to be 0.2811. Whereas, among the masked and segmented pixels, the registration exhibited a mean error of non-overlapping pixels of 0.2814.

### 3.7. Performance of YOLOv8l-Seg towards Prediction of Wild Radish Mask Instances

**Figure 12:**
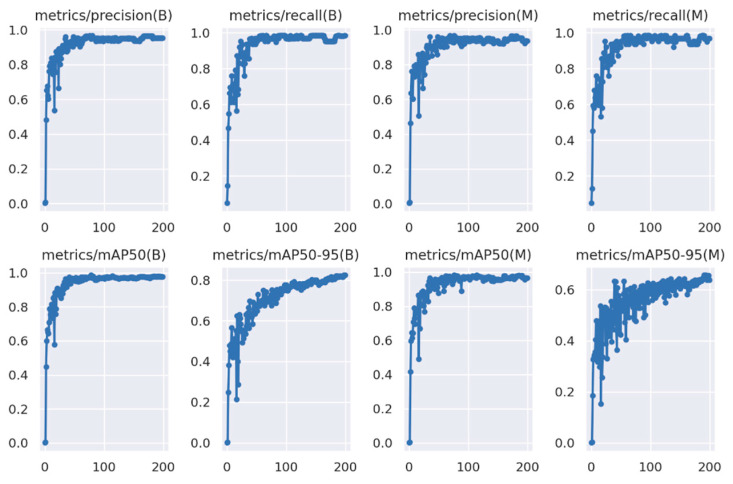
YOLOv8l-seg performance metrics.

**Figure 13:**
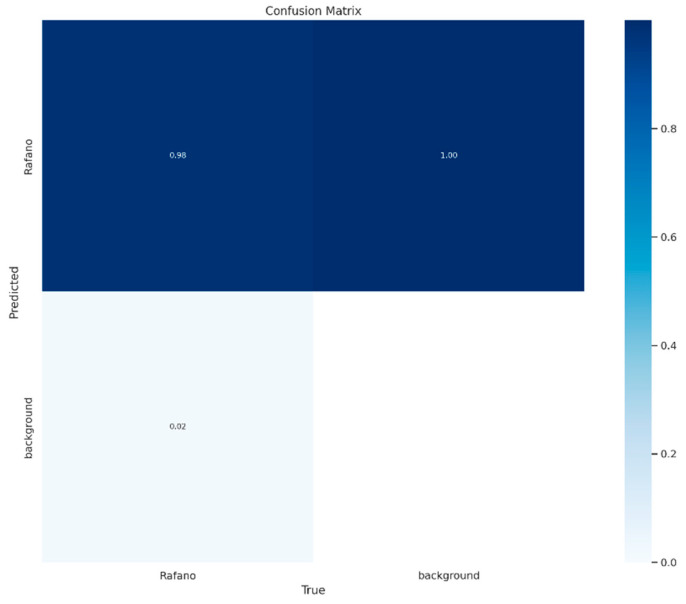
Confusion matrix of predicted wild radish labels.

**Figure 14:**
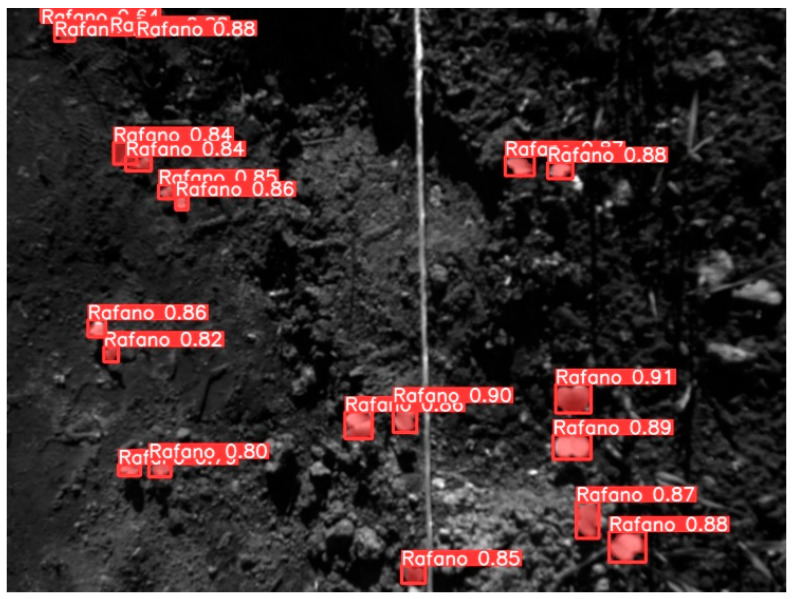
Wild radish instances predicted using YOLOv8l-seg.

YOLOv8l-seg demonstrated an effective segmentation approach towards annotated MS images where the wild radish instances were based on pixel annotations. Figure 12 represents the model’s performance metrics recorded post-completion of the training process. The mean precision (P) calculated at the bounding box level was recorded to be 0.9127. The mean recall (R) value calculated at the bounding box level was recorded to be 0.9278. The mean average precision (mAP50 or mAP0.5) at a 50% IoU threshold, considering both the wild radish class and the background class was recorded to be 0.9332. The mean average precision (mAP50-95) across 50% to 95% IoU thresholds, considering both the wild radish class and the background class was observed to be 0.706. However, the P, R, mAP50, and the mAP50-95 in the case of binary mask predictions were observed to be 0.9021, 0.916, 0.9211, and 0.554, respectively. The confusion matrix (Figure 13) demonstrates that 98% of wild radish labels were correctly predicted with the classifier (Figure 14).

## 4. Discussion

Our study aimed to comprehensively evaluate three MS image registration strategies for proximal remote sensing of weeds in close-ranged MS images. These strategies were focused on harnessing SIFT and RANSAC applied towards spatial realignment of pixel annotations, registration based on binary masks, and masked pixels. These techniques enabled the registration of close-range non-georeferenced MS images acquired with MicaSense RedEdge-M sensor over 16 consequent image scenes containing wild radish weed infestation among bread wheat crops. Every scene consisted of manual acquisition across five different spectra, summing up to 80 images in total. To facilitate weed detection and segmentation from MS images, the modus operandi solicited an optimized approach for effective alignment and overlap of annotations across all registered spectral channels towards integration of the spectral information to gain more complex and detailed scene representation [35].

The decision to use a perspective transformation over an affine transformation for image registration was based on the very nature of the images being registered where significant differences in perspective or viewpoint were observed. Using perspective warping, points from one plane were mapped to another to overcome projective distortions [40]. It was observed that perspective warping could also perform all the operations of an affine transformation plus account for distortions caused by changes in viewpoint or camera position.

Section 1 discusses the overall performance of three registration strategies and elaborates on the performance of each technique with a specific focus at the spectral level. Section 2 elaborates on the performance of these techniques at the instance level across different spectra. Section 3 highlights the performance of these techniques on automatically predicted instances of wild radish. Section 4 discusses the error observed in the form of non-overlapping wild radish instances across the three underlying registration strategies. The concluding section confers the performance of the deep learning model YOLOv8l-seg towards the generation of pixel-level masks for all the detected wild radish instances.

### 4.1. Overall Performance of Different Registration Strategies

It was observed that registration of MS images based on the registration of binary masks was overall recorded as the best-performing technique, securing the highest mean segmentation accuracy of 0.8314 and a mean normalized correlation coefficient of 0.9059 (Figure 10, Table 2, Section 3.1). The segmentation quality was observed to be the highest in the near-infrared channel, recording a mean segmentation accuracy of 0.8726 and a mean normalized correlation coefficient of 0.9170 (Table 3, Section 3.2). The highest cumulative segmentation accuracy of 0.8614 and cumulative mean normalized correlation coefficient of 0.9252 were observed across near-infrared spectral channel, making it the most suitable spectrum for annotation purposes (Table 3, Section 3.2). Moreover, the brighter appearance of vegetation observed in near-infrared and RedEdge spectra is due to the reflectance by chlorophyll that facilitates manual annotation. This behavior is particularly due to chlorophyll, which absorbs most of the light in the visible range but is transparent to light with wavelengths above 700 nm [63,64]. Therefore, the RedEdge spectrum was chosen as a reference for all registration methods.

### 4.2. Performance of Registration Strategies over Individual Instances across Spectra

The registration of individual MS wild radish instances based on the registration of corresponding individual binary mask instances was found to be the most accurate method for individual instance registration. A cumulative mean segmentation accuracy of 0.9124 and a cumulative mean normalized correlation of 0.9511 were observed across instances (Table 4, Section 3.3). The green channel also recorded the overall best performance as compared to other spectral channels, where individual wild radish instances were registered to secure a mean segmentation accuracy of 0.9339 and a mean normalized correlation of 0.9658. This second-best registration for MS instances was based on the masked wild radish pixels in the red channel, recording a mean segmentation accuracy of 0.9159 and a mean normalized correlation of 0.9573, thereby making it a categorical winner in the red channel. Individual instances derived from spatially realigned pixel annotations were also observed to exhibit a fair registration potential in the green channel. The least standard deviation was observed for registration performed in the red channel. Therefore, registration of MS images based on binary masks has shown a strategic edge over the other two registration methods even when individual pixel instances were registered instead of registering a binary mask image containing multiple instances.

### 4.3. Performance of Registration Methods over Predicted Instances across Different Spectral Channels

The MS image registration method based on the registration of predicted binary mask instances was observed to exhibit a mean segmentation accuracy of 0.7188 and a mean normalized correlation coefficient of 0.8099. This technique performed marginally better than the method based on the registration of predicted and masked pixels, where a mean segmentation accuracy of 0.7185 and a mean normalized correlation coefficient of 0.8016 were observed (Table 5, Section 3.4). Spectacularly, the blue channel was observed to exhibit the highest mean segmentation accuracy of 0.7481 and a mean normalized correlation coefficient of 0.8525 for the registration applied to predicted binary masks. The registration applied over predicted and masked pixels depicted the highest registration accuracy, particularly in the green channel, displaying a mean segmentation accuracy of 0.7378 and a mean normalized correlation coefficient of 0.8462. Therefore, the registration of MS images based on the registration of automatic mask instance predictions indicates a marginal superiority over the registration method based on masked and predicted pixels in terms of segmentation accuracy and normalized correlation behavior.

### 4.4. Error Evaluations across Registration Methods

The mean absolute error percentage of non-overlapping instances observed across the three registration strategies also demonstrated a marginal superiority of the MS image registration technique based on binary masks (Figure 11, Section 3.6). This performance was subsequently followed by the method involving registration based on masked pixels and subsequently, the registration based on the spatial realignment of annotations. The MS registration method based on the registration of automatically segmented binary mask instances demonstrated a marginally lower error percentage of non-overlapping pixels, again resulting in superiority over the registration method based on masked and predicted pixels. Additionally, the least number of outliers were observed for the MS image registration method based on the registration of binary masks (Figure 10, Section 3.1). In a nutshell, the lowest error percentage of non-overlapping instances was observed for MS image registration based on the registration of automatically segmented mask instances.

### 4.5. Automatic Prediction of Wild Radish Instances

The YOLOv8l-seg model’s accuracy for the prediction of wild radish mask instances was recorded to be 98%. There has been a recent study on heterogeneous MS UAV (unmanned aerial vehicle) image registration for cotton leaf lesion grading, which used the EfficientDet neural network for the detection of lesion-affected pixels in true color (RGB) images, followed by SIFT + template matching-based image registration for RGB and MS images to automatically segment cotton leaf lesion [65]. The prime difference in our work with respect to this mentioned study is three registration strategies using SIFT + RANSAC to achieve registration of MS images through (i) spatial realignment of annotations, (ii) registration of binary masks, and (iii) registration of masked pixels. There has been an extensive comparison at the spectral level and individual instance level, using the aforementioned registration approaches performed on manually and automatically segmented MS images. Additionally, the nature of the imagery in our case is close-range non-georeferenced MS images, which were acquired manually at the sub-meter level. Another work, found to be partially related to our experiment, was based on the registration of close-range and non-georeferenced MS images acquired in a greenhouse environment. This work compared spectral performances across different types of geometric transformations [24]. In contrast, our activity was conducted in field conditions. Moreover, our strategy was based on registration through annotation realignment, and registration of mask instances and masked pixels using SIFT + RANSAC. Another similar experiment by [7] claimed not to make any assumptions about the scene structure and only required dense matching between two spectrally similar bands, making it applicable to imagery captured from all types of scenes, regardless of their geometric or radiometric properties. However, it seemed that its scope lacked testing over absolute sub-meter nadir imagery in a heterogeneous field environment, where the challenge of perspective distortion arises. In contrast, these issues were the primary focus of our study and were addressed through strategies based on homographic refinement for sub-centimeter-sized agricultural weeds of interest.

**Figure 15:**
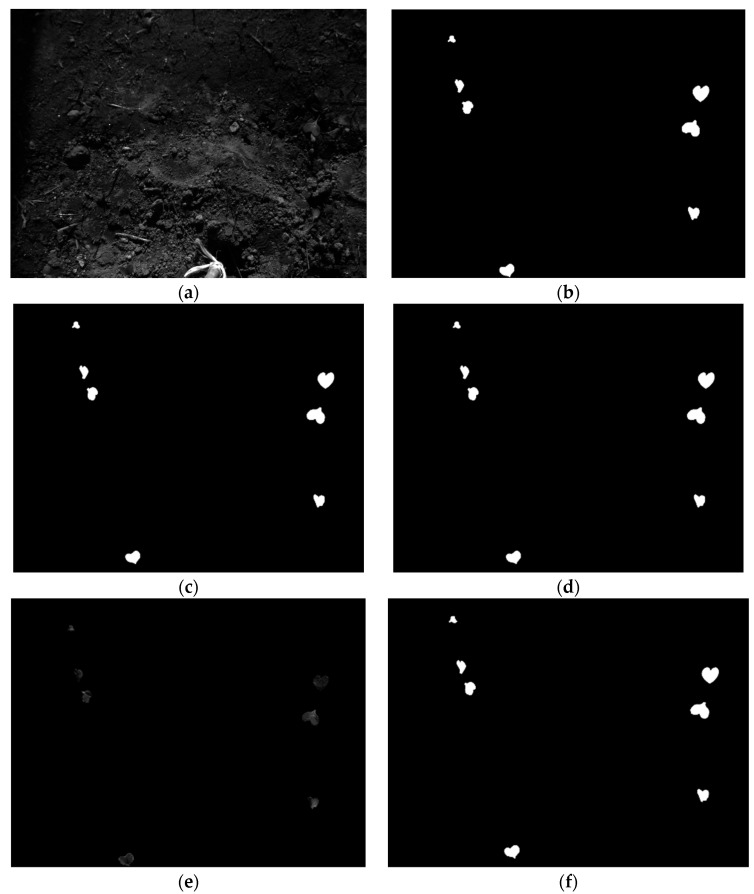

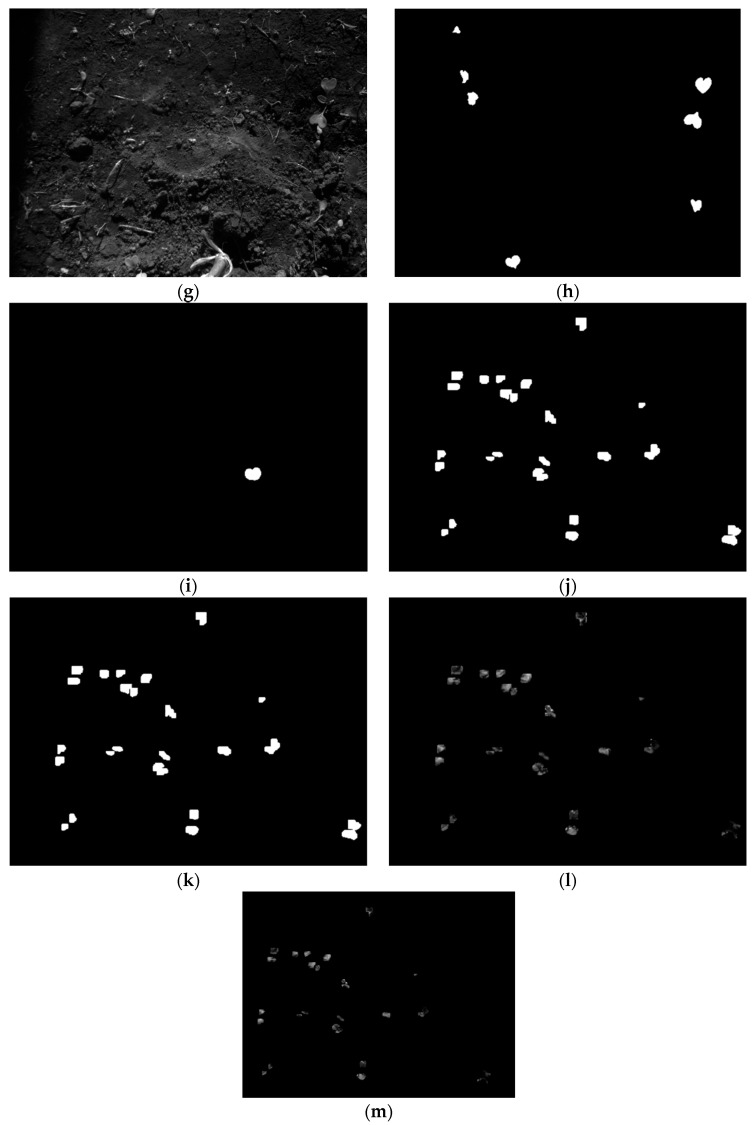
Few instances from the dataset. (**a**) Moving image (blue channel); (**b**) ground truth of moving image; (**c**) binary mask derived after spatial realignment of annotations; (**d**) registered binary mask; (**e**) pixels masked from ground truth; (**f**) binary mask derived from registered pixels; (**g**) reference image (RedEdge channel); (**h**) binary mask of reference image; (**i**) binary mask of one wild radish instance; (**j**) binary mask instances predicted with YOLOv8l-seg; (**k**) registered binary mask instances; (**l**) masked wild radish pixels (dilated); (**m**) masked wild radish pixels (registered).

Figure 15 is a categorical representation of the images containing a few spectral instances and binary masks derived from registration strategies explained in this article.

## 5. Conclusions

The manually acquired sub-meter images with a five-band MS camera face challenges of distortion in perspectives and altitudinal jitters leading to non-overlapping features. This article compared three image registration strategies based on SIFT and RANSAC algorithms over MS images containing a heterogeneous mix of bread wheat and wild radish. These MS image registration methods were based on (1) the application of a homography matrix derived during registration to realign the annotations across different spectral channels with respect to the reference channel, (2) based on the registration of binary masks derived from the ground truth of each spectral channel with respect to the reference channel, and (3) based on the registration of only the masked pixels of interest from each spectral channel with respect to the reference channel. The results show that the registration method based on binary masks is the most accurate, followed by the methods based on masked pixels and realignment of annotation. The accuracy also varies depending on the spectral channel and the segmentation technique used. This article also reports the performance of YOLOv8l-seg towards the detection and segmentation of wild radish weed instances. Additionally, it compares the aforementioned registration techniques over manually and automatically segmented images. In the future, this could be a possible part and parcel of a deep learning pipeline focused on automatic annotation, followed by automatic registration of MS images acquired in real time. The detection accuracy recorded with mean average precision at an IoU threshold of 0.5 was observed to be 92.11% with a segmentation accuracy of 98% towards the segmentation of wild radish binary mask instances.

## Figures and Tables

**Table 1:** Technical Specifications of MicaSense RedEdge-M.

Spatial resolution	0.07 cm/pixel at 1 m altitude
Frame rate	1 image/second
Wavelength (nm)	Red (668 nm), green (560 nm), blue (475 nm), near-IR (840 nm), RedEdge (717 nm)

**Table 2:** Mean values of different metrics for comparison of registration applied on annotations, binary masks, and masked pixels.

Method	Intersection over Union	Normalized Coefficient of Correlation
Registration based on spatial realignment of annotations	0.8055	0.8900
Registration applied on binary masks	0.8314	0.9059
Registration applied on masked pixels	0.8183	0.8971

**Table 3:** Registration quality across individual spectral channels.

	Cumulative Performance		Spatial Realignment of Annotations		Registration Based on Binary Masks		Registration Based on Masked Pixels	
Spectral Channels	IoU	NCC	IoU	NCC	IoU	NCC	IoU	NCC
Blue	0.8206	0.8996	0.8091	0.8929	0.8364	0.9094	0.8162	0.8965
Green	0.8408	0.9128	0.8266	0.9043	0.8476	0.9155	0.8481	0.9173
Red	0.7509	0.853	0.7415	0.8474	0.7690	0.8654	0.7421	0.8462
Near-infrared	0.8614	0.9252	0.8449	0.9155	0.8726	0.9170	0.8667	0.9284

**Table 4:** Mean values of IoU and NCC for registration applied on individual wild radish instances across all spectral bands.

Spectral Channels	Registration Based on Spatial Realignment of Annotations		Registration Based on Binary Masks		Registration Based on Masked Pixels	
	IoU	NCC	IoU	NCC	IoU	NCC
Blue	0.8962	0.945	0.8961	0.9453	0.8081	0.892
Green	0.8969	0.9457	0.9339	0.9658	0.7539	0.7539
Red	0.8894	0.9413	0.8872	0.9282	0.9159	0.9573
Near-infrared	0.8249	0.9046	0.9326	0.9652	0.8871	0.9407
Cumulative average	0.8768	0.9341	0.9124	0.9511	0.8412	0.8859

**Table 5:** Mean values of IoU and NCC for registration applied on predicted wild radish instances across all spectral bands.

Method	IoU	NCC
Registration based on predicted binary masks	0.7188	0.8099
Registration based on predicted and masked pixels	0.7185	0.8016

**Table 6:** Mean values of IoU and NCC for registration applied on predicted wild radish instances across individual spectral bands.

Spectral Channels	Registration Based on Predicted Binary Masks		Registration Based on Predicted and Masked Pixels	
	IoU	NCC	IoU	NCC
Blue	0.7481	0.8525	0.7171	0.8313
Green	0.7223	0.8357	0.7378	0.8462
Red	0.6723	0.8009	0.6751	0.8019
Near-infrared	0.7353	0.8456	0.7144	0.8298
Cumulative mean	0.7195	0.8336	0.7111	0.8273

## Data Availability

The data and the code used in this research are publicly available in the Zenodo repository. The link for accessing and downloading the dataset is https://zenodo.org/records/10567784 (accessed on 29 January 2024).
